# Experiences with and perspectives on advance care planning in young- and late- onset dementia: A focus group study with physicians from various disciplines

**DOI:** 10.3389/fnagi.2023.1130642

**Published:** 2023-03-28

**Authors:** Romy Van Rickstal, Aline De Vleminck, Sebastiaan Engelborghs, Lieve Van den Block

**Affiliations:** ^1^End-of-Life Care Research Group (VUB/UGhent), Brussels, Belgium; ^2^Research Foundation—Flanders (FWO), Brussels, Belgium; ^3^Department of Neurology, University Hospital Brussels, Brussels, Belgium; ^4^Center for Neurosciences, Vrije Universiteit Brussel, Brussels, Belgium

**Keywords:** advance care planning (ACP), young-onset dementia (YOD), focus group (FG), physicians, late-onset dementia

## Abstract

**Introduction:**

Despite the relevance of advance care planning (ACP) for people with dementia, its uptake in this population is particularly low. Several challenges for ACP in dementia have been identified from physicians’ perspectives. However, the literature available mainly includes general practitioners and focuses exclusively on the context of late-onset dementia. This is the first study to inquire physicians from four highly relevant specialisms in dementia care, with a focus toward potential specificities based on patients’ age. The research question of this study is: “What are physicians’ experiences with and perspectives on discussing ACP with people with young- and/or late-onset dementia?”.

**Methods:**

Five online focus groups were conducted with 21 physicians (general practitioners, psychiatrists, neurologists and geriatricians) in Flanders, Belgium. Verbatim transcripts were analyzed through the qualitative method of constant comparative analysis.

**Results:**

Physicians believed that the societal stigma related to dementia influences people’s reaction to their diagnosis, at times characterized by catastrophic expectations for the future. In this regard, they explained that the topic of euthanasia is sometimes addressed by patients very early in the disease trajectory. Respondents paid ample attention to actual end-of-life decisions, including DNR directives, when discussing ACP in dementia. Physicians felt responsible for providing accurate information on both dementia as a condition, and the legal framework of end-of-life decisions. Most participants felt that patients’ and caregivers’ wish for ACP was more driven by who their personality than by their age. Nonetheless, physicians identified specificities for a younger dementia population in terms of ACP: they believed that ACP covered more domains of life than for older persons. A high consistency regarding the viewpoints of physicians from differing specialisms was noted.

**Discussion:**

Physicians acknowledge the added value of ACP for people with dementia and especially their caregivers. However, they face several challenges for engaging in the process. Attending to specific needs in young-onset, in comparison to late-onset dementia, requires ACP to entail more than solely medical domains. However, a medicalized view on ACP still appears to be dominant in practice as opposed to its broader conceptualization in academia.

## Introduction

Advance care planning (ACP) is defined as a process of communication between patients, family caregivers and professionals to explore patients’ preferences for future (medical) care, including at the end of life (Sudore et al., 2017). The concept has evolved considerably over time, now focusing on an ongoing process that also helps prepare people for “in the moment decision making” when necessary, rather than focusing on the completion of advance directives (Van den Block, 2019; Tishelman et al., 2021). In general, dementia leaves people with a relatively long timeframe of loss of ability to self-manage care and diminishing cognitive function (Gaster et al., 2017). Despite ongoing discussions about the value of ACP (Tishelman et al., 2021), it is argued that ACP can be particularly relevant for people with dementia and their caregivers as the condition eventually precludes patients from taking part in their own treatment decisions (Alam et al., 2022). In case of Alzheimer’s Disease, diagnosis can be made during stages of mild cognitive impairment. The larger timeframe for planning care, due to earlier diagnosis, increases the opportunity for and importance of ACP (Porsteinsson et al., 2021). Nonetheless, the uptake of ACP in dementia is low with less than 40% of patients worldwide undertaking ACP (Sellars et al., 2019). Research showed that having dementia, in comparison to other conditions, is negatively associated with discussing treatment preferences, indicating that there are certain specific challenges related to engaging in ACP in dementia (Evans et al., 2014).

Particularly in dementia, discussing future care is considered difficult due to uncertainties regarding the future and due to the jeopardized decisional capacity of people with dementia (Tilburgs et al., 2018a; Sellars et al., 2019). More specifically, a recent meta-review of systematic reviews and primary studies (Keijzer-van Laarhoven et al., 2020) showed that physicians feel responsible for providing high-quality end-of-life care to people with dementia but face moral dilemmas that may cause them to behave avoidantly toward initiating ACP. Among others, these dilemmas arise from not wanting to emotionally burden patients, trying to maintain hope, dealing with uncertainties in patients’ prognoses and having ethical concern regarding patients’ declining capacity (Keijzer-van Laarhoven et al., 2020). Fearing a shift in patients’ preferences as the condition progresses was also identified as causing reluctance for physicians to make advance decisions with people with dementia (De Vleminck et al., 2014). Conversely, a qualitative study also found that beliefs about the perceived benefits of ACP can motivate physicians to engage people with dementia in the process, such as the belief that ACP would align patients’, family caregivers’ and clinicians’ care goals (Alam et al., 2022).

For a more inclusive understanding of physicians’ attitudes and challenges in terms of ACP in dementia, several physician specialties that are essential in dementia diagnosis and care should be inquired. Although there is literature available, these studies mainly include general practitioners and focus exclusively on the context of late-onset dementia (De Vleminck et al., 2014; Tilburgs et al., 2018b; Alam et al., 2022). There is a dearth of studies that inquire physicians from various specialisms. Moreover, research in which physicians are questioned about their perspectives not only regarding late-onset, but also young-onset dementia (YOD) is absent. Globally, it is estimated that 370,000 people younger than 65 develop dementia symptoms before the age of 65 annually, defined by the term YOD (Hendriks et al., 2021). The very limited number of studies focusing on people with YOD and their family caregivers, showed that they barely engage in ACP, yet have clear preferences for how to do so (Van Rickstal et al., 2019). Among others, these include their wish for physicians to timely initiate and flexibly approach the process, provide accurate information and pay attention to more than only the medical aspects of care (Van Rickstal et al., 2019, 2022).

To the best of our knowledge, this is the first inquiry of physicians from four highly relevant specialisms in dementia care (GP’s, psychiatrists, neurologists and geriatricians) regarding ACP, with a specific interest toward the potential specificities depending on patients’ age at diagnosis. The research question of this study is: “What are physicians’ experiences with and perspectives on discussing ACP with people with young- and/or late-onset dementia?”.

## Materials and methods

### Design

This exploratory study used the qualitative research method of focus groups, as this approach allows for open discussion and interaction between participants. Conducting focus groups online was necessary due to the COVID-regulations at the time yet was also an attempt to minimize participation burden for already challenged healthcare providers. In adherence with a recent guideline for virtual qualitative data-collection (Dos Santos Marques et al., 2021), the maximum participants per focus group was lowered (*n* = 5) to facilitate in-depth discussion. This paper follows the COREQ-criteria for reporting qualitative research.

### Participants

To answer our research question, we aimed for a heterogeneous sample in terms of physicians’ specialism within focus groups, to allow for in-depth insights. We included general practitioners, neurologists, psychiatrists and geriatricians as these specialties are crucial in the care for people with dementia. Physicians were purposively sampled through a personal email of the main researcher (RVR) or through a general recruitment mail spread within several organizations (Belgian Dementia Council, and the Flemish Associations for Psychiatry, Geriatrics, and Neurology). After physicians expressed their willingness to participate, they were sent a doodle in which they could indicate suitable moments for the focus group to take place.

### Data-collection

For these focus groups, an interview guide consisting of open-ended questions was developed within the research team (see Box 1). Participants were informed about some important “ground rules” at the start of each focus group, such as no talking across each other, respecting confidentiality regarding others’ participation, the content of discussions, etc. Each focus group was moderated and observed by two researchers (four by RVR and ADV, one by RVR, and LVdB). The focus groups took place online through secured Zoom-meetings in November and December 2021. The focus groups were conducted in Dutch, were video- and audiotaped with participants’ consent and were transcribed verbatim. After the fifth focus group, researchers reached consensus that data-saturation had been reached and no additional focus groups needed to be organized.

**BOX 1. Focus group topic guide.**

**1. Introduction**

Description of ACP provided by researchers:

*“Advance care planning is a process of communication between patients, their family caregivers and professionals in which patients’ views, values and preferences for future (medical) care are explored. This process should enable patients to help guide future decisions (also at those times when they are no longer able to make or express choices). ACP can, but does not necessarily, result in the documentation of wishes in advance directives”*

To what extent is this description similar to how you conceptualize ACP/your understanding of the concept?

**Throughout the following questions, respondents were systematically asked if there were any specificities in case of young- vs. late-onset dementia.**

**2. Experience with ACP**

**To what extent** do you engage in ACP in your clinical practice?

If you engage in ACP with patients/family caregivers:

   Who usually initiates the communication?
     If at physician’s initiative: How do you usually initiate ACP?

Is there, in your experience, **a right time** to initiate ACP?

**Who** is usually involved in ACP? (patients, family caregivers, other care professionals,…)

What are important **topics** to discuss within ACP?

Are there specific **hindering factors** when it comes to engaging in ACP in case of dementia?

**3. Wish to engage in ACP from patients/caregivers**

In your experience, to what extent do you feel there is a **need/wish for ACP** from patients and their family caregivers?

What is the **added value** of engaging in ACP in dementia? Is there a difference in this value, in your perspective, for patients vs. for family caregivers?

### Data-analysis

Verbatim transcripts of the focus groups were analyzed through the qualitative method of constant comparative analysis (Hewitt-Taylor, 2001; Dierckx de Casterlé et al., 2012). In this inductive approach, a code is assigned to a certain idea or concept (usually one or two sentences). These codes are subsequently compared within and between transcripts, identifying broader themes or concepts. Two transcripts were read and coded in full independently by two researchers. After discussion and agreement on a coding structure, the remaining three transcripts were coded and analyzed by RVR. Once coding was completed and codes were added to the coding framework, RVR and ADV together revised the transcripts and the obtained coding structure.

### Ethics

The study was approved by the Ethics Committee of the University Hospital Brussels (B.U.N. 143201939497) as the central commission and by Hospital Network Antwerp (ZNA, approval no 5208) and GasthuisZusters Antwerp (GZA, 190304ACADEM) as local commissions. A signed informed consent was obtained by all participants prior to the start of the focus group.

## Results

The average duration of a focus group was 95 min. A total of 21 physicians took part in one of five focus groups (two *n* = 5, two *n* = 4, one *n* = 3). Of these 21 physicians, five were general practitioners, three were specialized in psychiatry, six in neurology, and five in geriatrics. Except for one last-year neurology resident, all were board-certified specialists. Five women and 16 men participated.

Six major themes were identified from our data: (1) stigmatic image related to dementia as a specificity for ACP in this population, (2) physicians’ focus on specific end-of-life decisions when discussing ACP in dementia, (3) physicians feeling responsible for providing information on dementia and on the law regarding end-of-life decisions, (4) the age of patients and caregivers as an influence on the content of ACP, (5) physicians seeing more benefits of ACP for family carers, and (6) congruency between medical professions. Several of our findings are generally related to dementia as a condition and can therefore be interpreted as applicable to both the young- and late-onset variant.

### Stigmatic image related to the condition as a specificity for ACP in dementia

A factor that physicians believed to negatively influence patients’ fears and concerns about the future, was the stigma related to dementia. In this regard they discussed how the popular media is at times responsible for diminishing nuances in people’s image of dementia: the last phase of disease progression is portrayed as representative for the entire disease trajectory.

*“That one quickly thinks that it’s only about that last vegetative stage and that one would also end up there very soon etcetera. In the beginning, that’s something that strongly traverses those conversations. One doesn’t know that there are many years preceding that” (FG 24, 138–140).*

Despite patients’ initial expectations regarding their disease progression, physicians referred to people with dementia who, along the way, sometimes find their trajectory more manageable than initially expected. From their perspective, this posed a difficulty for engaging in ACP, since the evolution of patients’ wishes was felt to be too unpredictable to offer guidance for future care decisions.

*“If they say ‘I don’t ever want to be in a wheelchair,’ or ‘I always want to be able to feed myself,’ or something like that, then eventually, when push comes to shove, they don’t mind being wheeled around or they don’t mind that they’re being cooked for. So, it changes so much that it’s not fully predictable” (FG23, 170).*

Some physicians explained that the “catastrophic” image of dementia at times caused patients to drastically react to receiving their diagnosis and that they, and especially younger patients, quite impulsively expressed a wish for euthanasia the moment of or soon after hearing their diagnosis.

*“When disclosing the probable diagnosis or the results, people very often or at least several times show a catastrophic reaction and then they immediately start thinking about that last stage” (FG24, 146).*

*“Yes, and with people with young-onset dementia.” There are a few patients who at the moment of diagnosis nearly immediately say “okay, I have said that I want euthanasia in that case” (FG65, 89).*

This moment was said to be grasped by physicians as an opportunity for further exploration, explanation and broader discussion of preferences.

*“If you then assess ‘what motivates that (euthanasia) question?’ or ‘what is truly behind it?’*… *Then you actually arrive at a much broader framework of care planning that basically no longer entails what the initial question for euthanasia was, but more about care and planning and those things*…*” (FG24, 75/76).*

### Physicians’ focus on specific end-of-life decisions when discussing ACP in dementia

All participating physicians were familiar with the description of ACP provided at the beginning of the focus groups. However, it became apparent that physicians mostly elaborated on or re-directed the conversation to a specific aspect of ACP, namely to anticipatory end-of-life decision-making, such as DNR-orders (do not resuscitate) and euthanasia.

#### Physicians’ perceived motives behind euthanasia requests

According to our participants, the request for euthanasia was usually a request for something else in terms of future care. In most cases, it turned out to be the patient’s expression of a concern for which they sought guidance rather than an actual wish for euthanasia.

*“In many cases it turns out that it (euthanasia request) is about other concerns that can easily be addressed in a different way and then the question disappears” (FG24, 84).*

*“Actually they are not asking for euthanasia, they are asking the question ‘if I end up in circumstances that I don’t find dignified, are you still going to help me?”’ (FG43, 94).*

This was also explained by physicians through the motives on which they thought these patients’ comments on euthanasia or euthanasia requests were based. Participants mentioned that these could stem from agitation about what the future will bring, unwillingness to move to a residential care facility and fear of the unknown.

*“What is said frequently, is ‘Yes, if I would have to go to a nursing home, then I’m done. I don’t want to live like a vegetable. I’ve seen it with my mother or my father. Then, I would actually prefer euthanasia and I want you to write that down in my file like that”’ (FR44, 171).*

### Physicians felt responsible for providing information on dementia and on the legal framework of end-of-life decisions

Many physicians also felt that media had contributed to both the public’s awareness about euthanasia as an end-of-life option and had contributed to confusion about what is possible or impossible under Belgian law. Explaining patients about the legal framework was said to be an important task in clinical practice in terms of ACP.

*“So, a big part of the time or a big part of the energy goes out to just explaining what’s possible and what isn’t possible” (FG44, 186).*

Additionally, it was mentioned that providing information (in terms of for instance law or prognosis) could function as a care intervention itself.

*“I often notice that by discussing and explaining it (the legal framework) and by defining it, they sometimes find some peace already. That that request (euthanasia) sometimes stems from fear of the unknown and that informing them is at times already sufficient to find peace. That the questions then sometimes also fade away to the background” (FG 24, 72–74).*

According to our participants, patients tended to hold a “catastrophic” view of (young-onset) dementia, characterized by drastically declining functional and cognitive abilities. Driven by this alarming image, patients at times initiate ACP or euthanasia discussions according to physicians. In this regard, participants underscore a clear need for education in the sense of prognostic information.

*“If we get the question (euthanasia), it’s usually indeed a question for, yes*… *that has a whole lot to do with the stigma around dementia, I think. Many people regard someone with Alzheimer’s disease as someone who sits in a wheelchair, drooling, in a nursing home, as a figure of speech. But of course that’s not always the stage that everyone progresses to. So, I think that it’s important to educate a bit in terms of what the possible patterns and expectations can be” (FG43, 101–104).*

In terms of discussing prognosis, physicians explained they typically use “vague” terms and “averages” when describing a patient’s medical future. This manner of communication was based on both clinical uncertainty about the dementia trajectory according to participants, and physicians’ wish to safeguard patient’s hope and positive emotions.

*“General terms are averages: but I try to avoid making individual predictions” (FG44, 109).*

*“One of the biggest problems from my experience is that, often, we are also not honest toward our patients with dementia” (FG43, 171–172).*

Although patients’ image of dementia might be “catastrophic” at times and in need of nuanced information, some physicians emphasized that one cannot deny the inevitable negative aspects when going through the entirety of a dementia trajectory. Participants felt that these aspects are difficult to disclose openly to patients.

*“It doesn’t always have to be as bad as dying drooling in a nursing home, but well, the cases in which the older man, the grumpy old man becomes the endearing father, those are less frequent than the other story” (FG43, 202).*

*“If we take good care of them and place them in a decent nursing home, then they die of, well, what do they actually die of? Do they starve? Do they have a spontaneous fracture because they have been lying in bed for years?” (FG43, 176–177).*

They expressed that a longitudinal and trusting relationship between patient and physician increased their “openness and honesty” in terms of disclosing prognostic information, for instance about the speed of disease progression or expected difficulties ahead.

*“The way in which you get more concrete in terms of prognosis, that’s also an advancing insight. After the diagnosis, the progression, the first two years that always gives an indication of how quickly it could evolve” (FG65, 166–167).*

*“And you don’t name it with, yeah, terms that are hurtful, but yeah*… *sometimes we have known these people for years. Yes, then I dare to be honest about it (prognosis). I’m quite straightforward and the people who continue to come into consultations with me, are the ones who can tolerate that and even expect it” (FG43, 180–181).*

### The age of patients and caregivers influences the content of ACP discussions

It was noted that both people with young-, as well as people with late-onset dementia are heterogeneous groups. The extent to which people wish to engage in ACP was generally regarded as connected to who the patient was as a person, rather than associated to the patient’s age.

*“There are people, both among younger patients, but also among older patients, who are very set on their autonomy and from that perspective can also be very verbal and have a clear request for ACP or other things. Just as well, there are younger patients who would rather avoid that type of conversation” (FG24, 98–99).*

Although some physicians said that younger vs. older people with dementia are usually more “articulate,” “assertive,” and “have a higher need for control”, the majority of physicians saw an equal amount of younger and older patients wanting to discuss ACP. However, they noted that the life context of younger people, with younger children and spouses, might make their questions about the end of life more salient.

*“I can imagine that under those circumstances the questions about wishes for the end of life are much more prominently present and that one contemplates it much more at that age compared to at an older age. With these younger people, they (wishes for the end of life) will be brought up sooner or later” (FG65, 109–110).*

Several physicians talked about how YOD, in comparison to late-onset dementia, might lead to diminished acceptance of the diagnosis, higher grief and to more conflict within families, among others about financial matters.

*“Older people already let go of life a bit more and accept that there they are at a high age at which things will end sooner or later” (FG65, 81).*

*“There is also much more sadness of people with young-onset dementia, for so many good years lost” (FG65, 139).*

Respondents explained that caregivers at times had a higher wish to engage in ACP than patients. Examples provided were when patients did not grasp the implications of their condition, were no longer cognitively competent or when patients had expressed a death-wish to their caregiver, who wanted to discuss this further with the physician. Several physicians explained that during their consultations, caregivers of younger as opposed to older, people showed more tendency to bring up ACP.

*“Of course I have people who have no illness awareness, and especially in that case that question will arise through the caregiver. Especially if there is no awareness of illness, then it all appears very ‘far off’ for the patient, and that can be difficult at times” (FG65, 57–58).*

*“Yes, it regularly occurs that some type of death wish was expressed by the patient and that that actually is the impetus for the partner or the children to initiate that conversation. They often refer back to it like ‘you remember that you’ve said that, what do you actually mean by that?”’ (FG65, 64–65).*

Additionally, physicians explained that ACP discussions usually cover more domains in YOD due to the challenges the diagnosis brings along in multiple areas of patients’ and caregivers’ lives.

*“Evidently, with younger people there is often the difficulty of the partner still working, that the children are still young, still studying, at times still living at home, which actually complicates it even more. Then that is a broader conversation, because it becomes even more difficult with caregivers themselves, that conversation” (FG65, 135).*

*“If there are children who are still young and who, just to give an example, become scared of their father or mother, or where their relationship changes entirely. Or a professional situation, people who are still working. You simply come across many more problems, which obliges one to consider at least a mid-long timeframe” (FG44, 133–134).*

### ACP was believed to especially benefit family caregivers

Several advantages of ACP engagement were discussed by physicians, for the majority relating to family caregivers. ACP was told to lead to an ‘emotional relief’, less conflict and less suffering since family caregivers were enabled to fulfill their need to provide care to their loved-one.

*“And I think that for family it’s also very important to have that feeling like ‘we are doing well, we have done well”’ (FG24, 33).*

Physicians believed that both patients and caregivers would assess the care provided as more positive, due to ACP.

*“The bottom line is of course that people, the caregiver as well as the patient, will evaluate the care received more positively in the sense that they feel it is more closely aligned to what they wish” (FG24, 23).*

From patients’ perspective, physicians hypothesized that not wanting to be a burden to others might be a motivating factor ACP, aside from keeping their own best interest for the future in mind.

*“By some (patients) it is indeed addressed that they somehow do it (ACP) for the caregivers, but it’s not an ‘or-or story’, it’s a combination of how they themselves feel about it” (FG65, 188).*

Patients’ need and desire to take care of their family and ACP as a means to fulfill that need, was noted as well.

*“That’s also partly taking care of my children. That’s drafting a care plan, so that my children know that it’s okay what they do or not do with me” (FG43, 249).*

### Congruency between medical professions

There were no divergent themes when comparing between physicians from differing specialties. Moreover, there appeared to be a consensus amongst respondents that general practitioners are usually able to play a key role in ACP, due to their usually longstanding relationship with the patient and his/her family, and their professional context in which they are more likely to have frequent consultations with patients, possibly including home visits. It was noted that systematic sharing of ACP information between the various physicians involved in a patient’s care was desirable, yet that such information flow was not sufficiently common.

## Discussion

### Summary of results

This study shows that physicians believe that the societal stigma related to dementia impacts how people react to their diagnosis, including catastrophic expectations for their future. In this regard, they mentioned that the topic of euthanasia is at times addressed early in the disease trajectory by patients. Physicians themselves paid ample attention to actual end-of-life decisions, including DNR directives, when discussing ACP in dementia. As part of ACP, physicians felt it was their responsibility to provide accurate information on both dementia as a condition, and the legal framework of end-of-life decisions. Most participants felt that patients’ and caregivers’ wish for ACP was more driven by who they are as people than by their age. Physicians did identify specificities for a younger dementia population in terms of ACP: they believed that ACP covered more domains of life than for older persons. A high consistency regarding the viewpoints of physicians from differing specialisms was noted.

### Strengths and limitations

The main strength of this study is that it assembled focus groups heterogeneously in terms of specialisms crucial in dementia care, allowing for in-depth insights from and for various medical disciplines. Our research question focused on people with late-onset, as well as with young-onset dementia. This led to findings that are insightful for clinicians, when caring for this underexposed group. A limitation of this study is that we did not observe actual practices, but analyzed what respondents shared about these practices. Also, certain results might be less or not generalizable to other legal contexts besides those with physician- assisted dying laws. In this regard, however, we deem our results to be informative within the current internationally evolving landscape of physician-assisted dying legislation. Future comparative research in countries with varying legislative frameworks would be insightful for understanding the possible impact of the law on ACP and on ACP communication.

### Interpretation of findings

Physicians explained that, at times, they struggled with disclosing prognostic information due to clinical uncertainty characteristic to dementia. The difficulty or even inability to provide accurate prognostic information experienced by our participants, has also been reported by patients and family caregivers in different countries (Sellars et al., 2019). It has been shown that patients and family caregivers felt a distrust toward clinician’s mastery and knowledge of dementia (Groen-van de Ven et al., 2017). Physicians communicating openly to their patients about their uncertainty, might counter such feeling of distrust and contribute to a relationship of mutual confidence and trust. This could in turn facilitate ACP, as a sense of rapport was previously identified as a prerequisite for ACP in dementia by patients, their caregivers and general practitioners (Tilburgs et al., 2018b; Van Rickstal et al., 2019). Attending to not only patients’ and caregivers’ uncertainty in decision-making (Sellars et al., 2019), but also to that of physicians, might empower all parties when it comes to initiating ACP. Comparing our findings with existing literature, showed that there is an important commonality between barriers identified by professional caregivers on the one hand, and barriers identified by family caregivers and patients on the other.

Physicians explained that disclosing prognostic information might also be hampered by constraints they experience in openly and honestly communicating about disease progression. Although participants acknowledged that a dementia trajectory undeniably has negative elements, they simultaneously pleated for a more nuanced image of dementia, with a sometimes more steadily progression than expected or feared. Qualitative studies showed that people with dementia and their caregivers tend to oscillate between “wanting to know” and “not wanting to know” (Wawrziczny et al., 2016) and prefer to take it “one day at a time” (Van Rickstal et al., 2019; Keijzer-van Laarhoven et al., 2020). Additionally, people with late- and young-onset dementia and their caregivers have previously highlighted that, regardless of being diagnosed with dementia, there is still room for enjoyment (Dening et al., 2017; Van Rickstal et al., 2019). Moreover, previous research showed that focusing on the present as opposed to worrying about the future, is associated with experiencing fewer unmet needs and therefore is an effective coping strategy (Millenaar et al., 2018). Having a realistic and truthful view on the future, yet also allowing hope and positivity to co-exist with this, appears a useful balancing act to be undertaken by all those involved when engaging in ACP. When placing our finding in the context of findings with patients and caregivers, it appears that physicians’ moral threshold to engaging in ACP, also described in previous research (Keijzer-van Laarhoven et al., 2020), is at times justified. According to participants, the societal negative image that is related to dementia increases the need for realistic information provision. Physicians in our study described how patients at times demonstrate catastrophic reactions to receiving a diagnosis, also based on the common, stigmatic image of dementia. If grasped by physicians, these reactions might function as a steppingstone to discuss ACP more broadly, according to them.

In the current study, ACP was considered by physicians to be a means or an opportunity for people with dementia of fulfilling a caretaking role toward their family. It has been previously stated by patients and caregivers that if people with dementia undertake ACP, one of their main purposes is to take care of their loved-ones (Van Rickstal et al., 2022). The relational, as opposed to purely individual, nature of ACP appears evident from the viewpoint of all parties involved. As such, particularly in the context of dementia, a family- rather than a solely patient-centered approached to ACP could be desirable. As physicians also expressed that their wish to safeguard patients’ emotional wellbeing shapes their own behavior in terms of ACP, the previous idea of a mutual protective role between people with dementia and their family caregiver (Van Rickstal et al., 2022) could be extended from a dyad to a protective triad which also includes the professional caregiver.

Physicians identified specificities for the content of ACP in YOD. The desire for ACP was put forward by our respondents as person- rather than age- and/or generation- related, depending on someone’s personality. Nonetheless, several specificities in terms of age were mentioned. Young-onset dementia usually affects people in the prime of life, with possibly children still living at home, financial commitments, work, and at times caring for older relatives themselves (Withall, 2013; Draper and Withall, 2016). According to our respondents, ACP in YOD was indeed considered to cover a broader range of domains due to the plurality of life-areas affected by the condition. Additionally, if the topic arose, it was told to be more prominently present in consultations with younger as opposed to older patients and caregivers. The general hypothesis that younger people with dementia and their caregivers have a higher need for ACP due to a higher wish for autonomy (Koopmans et al., 2015), appears not to correspond with patients’, family caregivers’ (Van Rickstal et al., 2019, 2022) or professionals’ narratives. However, our former and current research shows that all parties do acknowledge that the content of ACP shows distinctions based on patients’ younger vs. older age, mainly due to stage of life. Through insights of patients with YOD and their carers it was previously recommended to conceptualize ACP as holistic (Van Rickstal et al., 2022), consistent with respondents of the current study who explain that ACP in YOD can entail a broader range of topics. Overall consensus seems to exist that clinicians need to dedicate heightened attention to non-medical domains to adequately address ACP in this younger population. However, it was formerly shown that Flemish people with YOD and their carers spontaneously incorporate euthanasia in their thought framework on end of life (Van Rickstal et al., 2020), and as such, it can also be regarded as a sensitivity from our participating physicians toward their patients that they pay adequate attention to end-of-life decisions. It appears recommended to find a balance between broadening ACP to medical, social and relational domains (Tilburgs et al., 2018b; Van Rickstal et al., 2022), yet simultaneously elaborating on specific concerns patients have, such as euthanasia, if this were the case.

## Conclusion

Overall, physicians acknowledge the benefits of ACP for people living with dementia and particularly for their family yet describe several challenges for actually engaging in the process. Some of these difficulties are related to dementia as a condition, others are associated with constraints for engaging in such conversations. Attending to specificities in terms of ACP for people with young-onset, compared to late-onset, requires physicians to pay attention toward non-medical domains. The finding that participants elaborated on actual end-of-life decisions, such as euthanasia and Do Not Resuscitate- directives, shows that the medicalized concept of ACP is still dominant in practice.

## Data availability statement

The datasets presented in this article are not readily available because we are unable to provide raw data as these include identifiable information from respondents (focus groups with physicians). Requests to access the datasets should be directed to RV, romy.van.rickstal@vub.be.

## Ethics statement

This study was approved by the Ethics Committee of the University Hospital Brussels (B.U.N. 143201939497) as the central commission and by Hospital Network Antwerp (ZNA, approval no. 5208) and GasthuisZusters Antwerpen (GZA, 190304ACADEM) as local commissions. The patients/participants provided their written informed consent to participate in this study.

## Author contributions

All authors listed have made a substantial, direct, and intellectual contribution to the work, and approved it for publication.
